# Mixed Germ Cell Tumor of the Endometrium: A Case Report and Literature Review

**DOI:** 10.1515/med-2020-0010

**Published:** 2020-02-04

**Authors:** Heping Zhang, Fangyun Liu, Jianguo Wei, Debin Xue, Zhengxin Xie, Chunwei Xu

**Affiliations:** 1 Department of Pathology, Fujian Cancer Hospital, Fujian Medical University Cancer Hospital, Fuzhou 350014, People’s Republic of China; 2 Department of Pathology, Anhui Province Maternal and Child Health Hospital, Hefei 230001, People’s Republic of China; 3 FenLan Medical Lab, Hangzhou 310052, People’s Republic of China; 4 Department of Pathology, Shaoxing People’s Hospital & Shaoxing Hospital of Zhejiang University, Shaoxing, People’s Republic of China

**Keywords:** Mixed GCTs, Extragonadal, Yolk sac tumor, Endometrium

## Abstract

Germ cell tumors (GCTs) localized extragonadally are rare, with only 14 reported cases of a yolk sac tumor in the endometrium. Here we report a case of mixed endometrium GCTs in a 65-year-old postmenopausal woman with abnormal vaginal bleeding. An ultrasound examination showed an oval-shaped mass in the patient’s uterine cavity. Biochemical examination revealed elevated serum α-fetoprotein (AFP) at 359 ng/mL, whereas the tumor markers CA-125, CA-199, and CEA were all within normal range. Total hysterectomy and bilateral salpingo-oophorectomy were performed;. a histological examination revealed that the malignant components contained a yolk sac tumor, embryonal carcinoma, and focal immature teratoma. Immunohistochemical staining showed that AFPs were diffusively distributed in both the yolk sac tumor and embryonal carcinoma. The stem cell marker OCT3/4 was positive in the embryonal carcinoma component and that the pan-cytokeratin AE1/AE3 staining was positive in glandular areas. GFAPs (Glial Fibrillary Acidic Proteins) were positive in neuroectodermal tubules; the Ki-67 protein was positive in 90% of the tumor cells, whereas CD117 and placental alkaline phosphatase (PLAP) were negative. The cumulative evidence indicated mixed GCTs of endometrium as the final histopathological diagnosis. The patient received three courses of adjunct chemotherapy that provided good therapeutic efficacy as evidenced by the decreased serum AFP level. Our report on this rare case of mixed GCTs of the endometrium, supported by associated histological patterns and immunophenotypes and successful adjunct chemotherapy after surgery, could provide insight on future treatment of this rare but lethal disease.

## 1 Introduction

Germ cell tumors (GCTs) include a number of histologically distinctive tumor types that are derived from the primitive germ cells of the embryonic gonad [1]. They frequently occur in the gonads (ovary or testes) of young people. Some GCTs are classified as extragonadal if there is no presence of primary tumors in either the testes or ovaries [2]. GCTs typically arise in midline locations along which the primitive germ cells migrate from the wall of the yolk sac to the gonadal ridge. In adults, the most common tumor sites are the mediastinum, retroperitoneum, sacrococcygeal regions, pineal glands, and suprasellar regions [3]. Mixed germ cell tumors are tumors having two or more types of malignant, primitive, or germ cell components; they represent about 8% of malignant GCTs. GCTs with a mixture of yolk sac tumor and dysgerminoma are the most common subtypes [4]. Primary GCTs arising in endometrium are extremely rare. Only 14 cases of primary yolk sac tumor of the endometrium have been reported [5, 6, 7, 8, 9, 10, 11, 12, 13, 14, 15, 16, 17] (Table 1). Here we report the first case of a mixed germ cell tumor located in the endometrium of a postmenopausal woman, including the clinical, histopathological and immunophenotypic features. Adjunct chemotherapy after surgery, which demonstrated an efficacious clinical outcome, is also reported as a promising approach for the treatment of the disease.

## 2 Case Report

**Figure 1:**
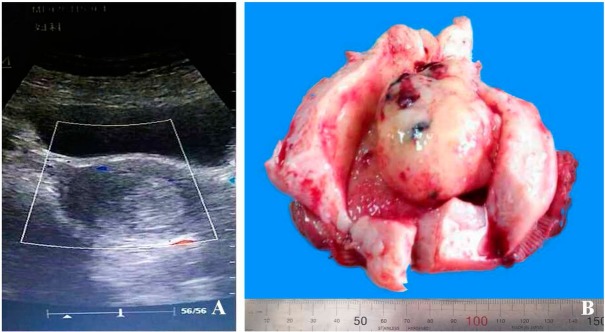
Imaging and gross feature of the case. A) Ultrasound images showing that the uterine cavity was occupied by an oval shaped mass. B) The gross appearance of the brown polypoid tumor located in the uterine cavity and originated from the endometrium.

A 65-years-old female, gravida 2 para 2, was admitted with abnormal vaginal bleeding that had continued for 2 months. A pelvic ultrasound showed that the uterine cavity was occupied by an 7.0×4.5 cm oval shaped mass; there was vascularization [Fig. 1A] but extensive examination for metastases that included a chest X-ray and thoracic computerized tomography (CT) showed no abnormalities. Biochemical examination revealed that tumor markers CA-125, CA-199, and CEA were all within normal range. However, her serum alpha fetoprotein (AFP) was elevated, at 359 ng/ml. Cytology of the cervix was negative for intraepithelial lesion or malignancy (NILM). The patient had a one-year history of diabetes that was being treated with metformin. Judged from those clinical features and imaging findings, a gynecologist concluded that it was likely that endometrial cancer was present. Therefore, curettage of endometrium was performed immediately. However, the pathological analysis revealed only a small amount of endometrial atrophy because of limited tissue samples. Then, total hysterectomy and bilateral salpingo-oophorectomy were performed.

### 2.1 Gross examination

The removed uterus measured 12.0×8.0×5.5 cm and weighed 185 g. Sectioning revealed that the depth of the uterine cavity was 10 cm, in which a brown polypoid tumor 7 cm in diameter was found. The tumor originated from the endometrium and extended into the myometrium; the depth of the invasion was 0.2 cm and the total myometrial thickness was 2.0 cm. The tumor was well circumscribed but non-encapsulated, with a brown soft cut surface and large areas of hemorrhage and necrosis. No hair and teeth were present. The thickness of the rest of the endometrium was 0.1 cm [Fig. 1B]. The appearance of the cervix was normal, with length 2 cm and diameter of 2.5 cm. The uterine serosa, bilateral ovaries and fallopian tubes, abdominal and pelvic wall and omentum had no unusual features.

### 2.2 Microscopic examination

**Figure 2:**
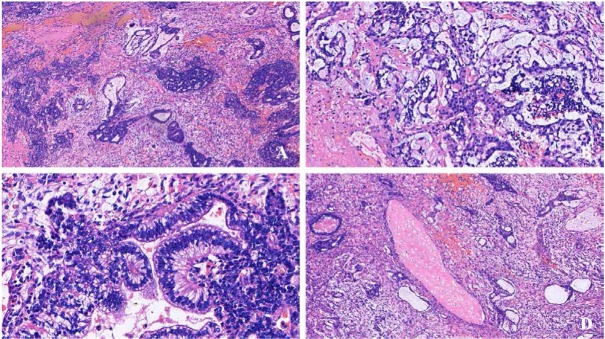
Histology of the mixed germ cell tumor. A) Low power photomicrograph showing mixed GCT with yolk sac tumor and embryonal carcinoma components (magnification: ×100). B) A representative histological image of reticular and microcystic architectures of yolk sac tumor components (magnification: ×400). C) A representative histological image of embryonal glands lined by columnar cells (×400). D) A representative histological image of mature teratoma in the form of mature bone tissue within the tumor (magnification: ×100).

Various morphological patterns were revealed by microscopic examination. A yolk sac tumor was the predominant component of the specimen and accounted for 40% of the entire tumor. The tumor cells were arranged in reticular and microcystic architecture. The stroma was loose and myxoid [Fig. 2A, B]. The second most predominant component (accounting for approximately 30%) was embryonal carcinoma, in which the tumor cells grew in solid, sheet, or nest patterns with gland differentiation. The solid pattern showed smaller polygonal cells with scant cytoplasm. The polygonal cells had vesicular nuclei with coarse, basophilic chromatin and one or two prominent nucleoli. Embryonal glands were visible, and the glands were lined with columnar cells [Fig. 2A, C]. Based on histological features, it was concluded that immature teratoma (Grade 1) comprised only a small portion of the specimen (approximately 5%). The immature teratoma included primitive neuroectodermal tubules lined by overlapping, hyperchromatic cells with numerous mitoses. Moreover, mature teratoma, which appeared in the form of benign mucinous glands and mature bone tissue, composed the rest of the tumor (approximately 25%) [Fig. 2D]. Large areas of necrosis and hemorrhage were observed. The tumor also showed local infiltration of the myometrium. However, no tumor was found in the cervix, bilateral ovaries, or fallopian tubes.

### 2.3 Immunohistochemical findings

**Figure 3:**
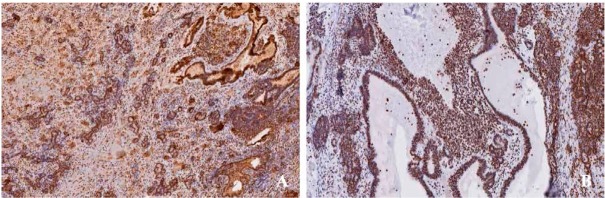
Immunophenotypes of the mixed germ cell tumor. A) Immunohistochenmical images showing that tumor cells were all positive after staining with monoclonal anti-AFP antibody (magnification: ×100). B) A representative image showing that OCT3/4 was positive in components of embryonal carcinoma (×100).

Immunohistochemical studies revealed that AFPs were diffusively distributed in both yolk sac tumor components, that the embryonal carcinoma component [Fig. 3A], OCT3/4 was positive in embryonal carcinoma component [Fig. 3B], and that AE1/AE3 was positive in glandular areas. GFAPs were positive in the neuroectodermal tubule component. Ki-67 was positive in 90% cells, whereas CD117 and PLAP were negative in all tumor cells. Combined with the observed clinical aspects, histological features, and immunohistochemistry results, the final diagnosis was mixed germ cell tumor of the endometrium, with the predominant malignant components being a yolk sac tumor (accounting for 40%), an embryonal carcinoma (accounting for 30%), and focal immature teratoma (accounting for 5%); the remaining components were benign mature teratomas.

Ethical approval: The research related to human use has been complied with all the relevant national regulations, institutional policies and in accordance the tenets of the Helsinki Declaration, and has been approved by Anhui Province Maternal and Child Health Hospital review board or equivalent committee.

Informed consent has been obtained from patient included in this study.

## 3 Discussion

Primary GCTs of extragonadal origin comprise 3% to 5% of all GCTs. They usually arise from midline structures but can also be found in three regions, such as the vulva, arm, and prostate [2,18, 19]. The histogenesis of extragonadal GCTs remains largely unknown. One hypothesis is that extragonadal germ cell tumors derive from primordial germ cells that fail to complete the normal migration along the urogenital ridge to the gonadal ridges during embryonal development. This erroneous process may stem from an abnormality in the primordial germ cell itself or dysregulation by microenvironment [20]. Another hypothesis is that primordial germ cells may migrate aberrantly during embryogenesis and undergo malignant transformation, ultimately giving rise to extragonadal GCTs.

Primary GCTs of the endometrium are extremely rare. To our knowledge, only 14 cases of yolk sac tumor in endometrium have been reported. Among the reported cases, 11 cases were pure yolk sac tumors and 3 cases coexisted with endometrial carcinoma. The age of patients ranged from 24 to 65 years (average, 40 years). Most patients showed abnormal vaginal bleeding and an elevated serum AFP. Other symptoms included abdominal pain, vaginal water discharge, and menorrhagia. In our patient, a postmenopausal woman, who although older than those cases previously reported, the clinical features are comparable, i.e., abnormal vaginal bleeding and elevated serum AFP.

The grossly exophytic or polypoid mass were located in the uterine cavity or cervical canal and were well circumscribed but non-encapsulated. The tumor size ranged from 1.3 cm to 10.5 cm. The cut surface of tumor was soft, fleshy, dark brown in color with some hemorrhage and necrosis. Histological findings in the present case were different from those previously reported, which usually showed typical characteristics of yolk sac tumor. Our case showed a mixed germ cell tumor, FIGO staging 1A. The predominant component was a yolk sac tumor, characterized by reticular, microcystic architecture with a loose and myxoid stromal. The second most predominant component was embryonal carcinoma, which showed solid, sheet, or nest patterns with gland differentiation. The tumor cells are smaller, polygonal with scant cytoplasm, vesicular nuclei, and coarse, with basophilic chromatin and one or two prominent nucleoli. Primitive neuroectodermal tubules, the typical features of immature teratoma, were also observed in focal areas. Immunohistochemistry is important in the diagnosis of mixed GCTs. AFP and OCT3/4 are the characteristic tumor makers for GCTs. AFP was positive in all yolk sac tumor and most embryonal carcinomas, whereas OCT3/4 was often expressed in embryonal carcinomas but absent in yolk sac tumors. In our case, both yolk sac tumor and embryonal carcinoma components showed diffused AFP expression. OTC3/4 was positive in embryonal carcinoma, but negative in the yolk sac tumor. CD117 and PLAP were also sensitive markers of GCTs, especially in dysgerminoma; however, in our case both of those two markers were negative for tumor cells, indicating no dysgerminoma. GFAP is a useful marker for glioma and was also positive in neuroepithelium. In our case, focal areas showed tubules lined by overlapping, hyperchromatic cells that were positive for GFAP. These cells indicated that the tubules originated from neural ectoderm.

The differential diagnosis of the mixed GCTs includes pure GCTs, clear cell carcinoma, and endometrioid carcinoma. Pure GCTs often contain a single component of primitive germ cell tumor, but mixed GCTs contains more than one germ cell tumor components. Hence, careful observation of the slides is critical to avoid missing small but clinically significant germ cell tumor components. In particular, when mixed GCTs show diffused areas of yolk sac tumor with massive microcystic and tubulopapillary patterns, it is important to distinguish them from clear cell carcinoma. This is because clear cell carcinoma of the endometrium also has various pathological patterns, including microcystic, papillary, solid, with predominantly clear or acidophilic cytoplasm. Whereas clear cell carcinoma cells express epithelial markers such as cytokeratin (CK) and epithelial membrane antigen (EMA), yolk sac tumor markers are different. Differential diagnosis of the disease also demands accurate determination of endometrioid carcinoma, the most common malignant tumor of endometrium; it causes abnormal vaginal bleeding or discharge and often occurs in elderly women. Endometrioid carcinoma is typically characterized by a glandular or villoglandular architecture lined by stratified columnar epithelium with crowded, complex branching architecture; it sometimes shows a solid growth pattern, whereas germ cell tumors show irregular glandular architecture in a loose or myxoid stroma, resembling endometrioid carcinoma with invasive growth. However, GCTs abundantly express germ cell markers, serum AFP in particular: this is the major difference between GCTs and endometrioid carcinoma. Moreover, a small number of cases of germ cell tumor of endometrium coexist with endometrial carcinoma [10, 11, 14], especially in elderly women. Therefore, it is of critical importance to identify all tumor components to differentiate GCTs and pure germ carcinoma, and to precisely tailor treatment for individual patients.

Similar to yolk sac tumor, GCTs have nearly always been fatal regardless of the primary site of tumorigenesis. However, over the last two decades, advances in diagnosis imaging, surgery, radiotherapy, and adjunct chemotherapy have produced significant improvements in the survival rate of patients with malignant GCTs. A combinatory treatment regimen of bleomycin, etoposide, and cisplatin (BEP) has shown great promise in the treatment of advanced and localized GCTs [21]. The 14 cases previously reported of yolk sac tumor of endometrium were all treated with surgery followed by chemotherapy, except for one patient who refused chemotherapy and three patients who also experienced radiotherapy. The follow-up times range from 8 months to 72 months (24 months in average). Metastasis was found in 6 patients, and the site of metastasis included pelvis lymph nodes, liver, lung, peritoneum, ovary, vagina, diaphragm, and vertebrae. Three patients died and two survived, for an overall survival rate of 11/14 (78.6%). In our case, the patient received three courses of BEP after surgery. A good therapeutic efficacy was observed, and serum AFP decreased to a normal level after 3 courses of therapy. The patient remained free of related diseases for 15 months after completion of the therapy.

## 4 Conclusion

We report a rare case of an extragonadal mixed germ cell tumor of endometrium in a postmenopausal woman. The predominant malignant components were yolk sac tumor, embryonal carcinoma, and both immature and mature teratoma. Immunohistochemistry staining was used to assist identification of each component. Adjunct chemotherapy after surgery demonstrated good therapeutic efficacy. Further studies are essential to characterize clinical features of this rare disease, to assist differential diagnosis, and to develop effective therapeutic regimens.
